# Fabrication Methods of Continuous Pure Metal–Organic Framework Membranes and Films: A Review

**DOI:** 10.3390/molecules29163885

**Published:** 2024-08-16

**Authors:** Qinglei Xing, Xiangyou Xu, Haoqian Li, Zheng Cui, Binrui Chu, Nihao Xie, Ziying Wang, Peng Bai, Xianghai Guo, Jiafei Lyu

**Affiliations:** 1 Department of Pharmaceutical Engineering, School of Chemical Engineering and Technology, Tianjin University, Tianjin 300350, China; 2 Key Laboratory of Systems Bioengineering, Ministry of Education, Tianjin University, Tianjin 300350, China; 3 Department of Catalytic Science and Engineering, School of Chemical Engineering and Technology, Tianjin University, Tianjin 300350, China; 4 Tianjin Key Laboratory for Marine Environmental Research and Service, School of Marine Science and Technology, Tianjin University, Tianjin 300072, China; 5 Key Laboratory of Ocean Observation Technology of Ministry of Natural Resources, School of Marine Science and Technology, Tianjin University, Tianjin 300072, China

**Keywords:** pure metal–organic framework membrane, membrane fabrication, separation

## Abstract

Metal–organic frameworks (MOFs) have drawn intensive attention as a class of highly porous, crystalline materials with significant potential in various applications due to their tunable porosity, large internal surface areas, and high crystallinity. This paper comprehensively reviews the fabrication methods of pure MOF membranes and films, including in situ solvothermal synthesis, secondary growth, electrochemical deposition, counter diffusion growth, liquid phase epitaxy and solvent-free synthesis in the category of different MOF families with specific metal species, including Zn-based, Cu-based, Zr-based, Al-based, Ni-based, and Ti-based MOFs.

## 1. Introduction

Metal–organic frameworks (MOFs) are a class of highly porous, crystalline materials [1], which have garnered significant attention in recent years due to their unique properties and potential applications [2]. These materials are constructed from diverse metal ions or clusters coordinated to multi-dentated organic ligands, creating a vast array of structures with tunable porosity, large internal surface areas, and high crystallinity [3]. The intrinsic porous architecture and versatility to customization have made MOFs suitable for a wide range of applications, including gas adsorption and storage [4,5,6,7], separation [8,9], catalysis [10,11,12,13,14], sensing [15,16,17], and drug delivery [18,19,20,21].

In comparison to the traditional polymeric separation membranes, the visibility and designability of the periodic pore geometry endow MOFs as fascinating candidate materials for membrane separation. The preparation of continuous and uniform MOF membranes enables applications that are unattainable with powdered materials, such as gas separation and seawater desalination. The processability of MOF thin films/membranes on various substrates has opened new avenues for integrating these materials into devices and systems where their unique properties can be exploited in confined geometries or at interfaces. However, MOF materials typically crystallize from the synthetic solution in powdered form, making it challenging to directly use them as membranes [22]. In addition, by embedding MOF particle as fillers into the polymer matrix [23], the mixed matrix membranes (MMMs) have encountered issues with poor compatibility between MOF fillers and the matrix [24]. In some cases, MOF-based MMMs prepared by the conventional blending and casting approach still demonstrate undesired microstructures, including “sieve-in-cage” defects, filler agglomeration, and pore plugging, which will undermine the membrane performance and mechanical properties of MMMs, causing them to rapidly deteriorate under relatively high MOF loadings [25]. Taking advantage of the ample porosity and periodic network of MOF materials, fabricating pure MOF membranes on porous substrates is anticipated to result in simultaneously enhanced permeability and selectivity, thereby disrupting the traditional trade-off balance of polymer membranes. The facile fabrication of pure MOF membranes has been investigated since the manganese (II) formate [Mn(HCO_2_)_2_] metal–organic framework film was first reported by the Caro team in 2007 [26]. Afterward, there has been an increasing amount of research attempting the preparation of pure MOF membranes to enhance the efficiency (flux and selectivity) of gas separation [27], azeotropes separation [28], organic solvent dehydration [29], and seawater desalination [30].

Due to the diverse constitutions and syntheses of different MOF families, it is impractical to prepare pure MOF membranes in a universal way. Several methods are currently being investigated for preparing pure MOF membranes, including conventional in situ solvothermal/hydrothermal growth [31], secondary growth of MOF seeds, electrophoretic deposition, counter diffusion growth, liquid phase epitaxy, and solvent-free synthesis. In the past few years, many excellent reviews have summarized advanced preparation strategies and applications of pure MOF membranes and mixed matrix membranes [23,32,33]. Previous reviews have focused on specific MOFs or MOF families; a comprehensive summary of the development of preparation methods for MOF membranes with various constituents has not yet been provided. Here, we summarized the preparation methods of pure MOF membranes, including in situ solvothermal growth, secondary growth, electrophoretic deposition, counter diffusion growth, liquid phase epitaxy, and solvent-free synthesis. Subsequently, the research about pure MOF membranes and films are comprehensively reviewed in the category of different metal species including Zn-based, Cu-based, Zr-based, Al-based, Ni-based, and Ti-based MOFs.

## 2. Fabrication Method of Pure MOF Membranes

### 2.1. In Situ Solvothermal Growth

**Figure 1:**
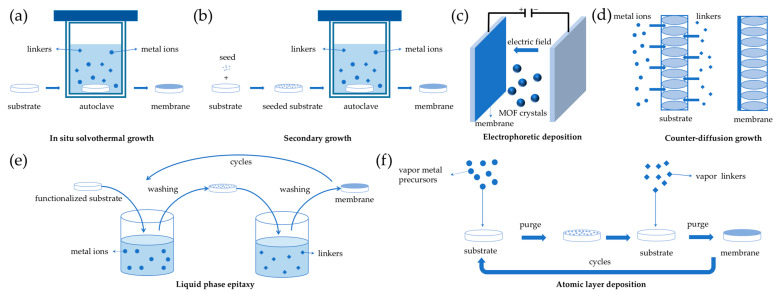
Schematic diagram of MOF membrane preparation method principle: (**a**) In situ solvothermal growth. (**b**) Secondary growth. (**c**) Electrophoretic deposition. (**d**) Counter diffusion growth. (**e**) Liquid phase epitaxy. (**f**) Atomic layer deposition.

In situ solvothermal growth represents a pivotal technique in the fabrication of MOF membranes, utilizing conditions similar to those employed in the crystallization of MOF particles. This method involves submerging porous supports into a precursor solution containing metal ions and organic ligands, which initiates heterogeneous nucleation on the support surface and facilitates the subsequent growth of MOF layers (Figure 1a). Since the pioneering work on the MOF-5 membrane on a porous alumina support achieved continuous MOF polycrystalline membranes [31], the in situ solvothermal growth has found broad applicability to various MOF membranes, such as UiO-66 [34] and ZIF-8 [35]. Despite the convenience, in situ solvothermal growth suffers from a low nucleation efficiency, difficulty to manipulate the thickness and orientation of the film, large consumption of reagents, and unavoidable homogeneous nucleation.

### 2.2. Secondary Growth of MOF Crystal Seeds

Fabricating pure MOF membranes by the secondary growth of MOF crystal seeds involves a pre-coated seed layer on the substrate as nucleation sites for further crystal growth. Subsequently, the seeded substrate is submerged in a growth solution containing metal precursors and organic ligands, where a solvothermal or hydrothermal reaction facilitates the formation of a dense MOF membrane (Figure 1b) [36]. Compared to in situ solvent thermal growth, secondary growth involves an additional seed layer preparation process, which increases the nucleation density for MOF membrane growth to a continuous and dense layer and enables membrane orientation control.

Various seeding methods have been identified to facilitate secondary growth. Manually rubbing involves the transfer of the seed crystal dispersion solution onto the substrate, followed by uniform distribution through applying it by hand [37]. Using the spin-coating method, a spinning coater is employed to distribute the seed solution evenly across the substrate [29]. The dip-coating method involves immersing the substrate into the seed solution and then drying to evaporate the solvent [38]. The reactive seeding method involves the reaction between metal sources on the substrate and organic ligands to strengthen the interaction between the substrate and the MOF layer [39]. The thermal seeding method involves dispersing the seed solution as well as metal species and the ligand solution onto the hot substrate surface [40].

### 2.3. Electrochemical Deposition

Electrochemical deposition (ECD) emerges as a versatile and effective technique for the fabrication of MOF membranes, which can be subdivided into anodic deposition [41], cathodic deposition [42], and electrophoretic deposition [43] according to the different mechanisms. In the anodic deposition process, the metal ions are provided by dissolving the metal anode through a voltage, which subsequently react with ligands in the solution to form a MOF film [44]. Cathodic deposition is typically carried out using a solution containing an organic ligand and metal precursor where the electrochemical reduction in anions (NO_3_^–^) results in an increased pH level around the cathode, promoting the deprotonation of organic ligands to form MOF films on the cathode surface [45]. Due to the defects in MOFs (e.g., missing linkers and metal nodes) and the free functional groups in linkers, the surface of MOF particles can become charged. Electrophoretic deposition capitalizes on the movement of charged MOF particles towards an electrode with an opposite charge when an electric field is applied across two conducting electrodes immersed in a MOF-containing solution (Figure 1c) [43].

The ECD process enables the rapid synthesis of continuous and high-quality MOF films at room temperature using simple experimental equipment, while also allowing for the control of film thickness by varying the voltage and current [33]. However, the correspondence between the metallic electrode and the metal centers in the resulting MOF films is required for anodic deposition. For cathodic deposition, the impurity of the metallic structure (originating from cathodic deposition of metals) is hard to avoid. The electrophoretic deposition of MOF particles is a viable coating technique to deposit various MOFs for sensors and catalysis, which will be the Achilles’ heel for separation due to the unavoidable voids and cracks [46].

### 2.4. Counter Diffusion Growth

Counter diffusion growth is the process of utilizing the metal ion solutions and ligand solutions on both sides of a porous substrate to diffuse in opposite directions, ultimately forming a MOF layer on the surface of the substrate (Figure 1d) [47]. The counter diffusion synthesis method is easy to operate and the reaction can be self-terminated while dense MOF layers gradually form with a high quality [48]. The crystal grains of the membrane can grow on the surface and in the pores of the substrate simultaneously, which improves the contact between the membrane and the substrate [49]. However, the counter diffusion synthesis method has been limited for the preparation of ZIF membranes, due to their mild reaction conditions [50].

### 2.5. Liquid Phase Epitaxy

Liquid phase epitaxy (LPE), also known as layer-by-layer (LBL) growth, involves alternately immersing the substrate into a metal precursor solution and an organic ligand solution, allowing the deposition of a MOF film on the surface of the substrate (Figure 1e) [51]. In order to better anchor metal ions and organic ligands onto the substrate, the functionalization of the carrier is usually required, with –COOH and –OH being mostly used as functional groups.

LPE enables the precise control of the orientation and thickness of the film through layer-by-layer deposition [52]. However, this process is cumbersome and consumes a large amount of solvent, which increases costs and causes environmental pollution [53]. More importantly, the LPE process is typically carried out at room temperature, making it unsuitable for MOFs with harsh synthesis conditions [23].

### 2.6. Solvent-Free Synthesis

The aforementioned methods often require a significant number of solvents, resulting in environmental pollution and resource waste. In order to achieve the goals of environmental sustainability and economic efficiency, the development of the solvent-free synthesis method is of great significance. MOF membranes have been prepared by solvent-free reactions between metal precursors (such as metal oxides) and organic ligands in gaseous [54], solid [55], or melted states [56]. Atomic/molecular layer deposition (ALD/MLD) has been developed for the direct gas-phase fabrication of MOF thin films through sequentially exposing the substrate to different precursor vapors and depositing single-layer atoms in each cycle (Figure 1f) [57]. Inert gas purging is needed to prevent the reactions between the precursors. ALD/MLD is beneficial for preparing ultra-thin MOF membranes, but it is limited to ZIFs with vaporizable ligands.

## 3. Fabrication of Zeolitic Imidazolate Framework Membranes

Zeolitic imidazolate frameworks (ZIFs) are porous crystal materials composed of divalent transition metal ions (Zn^2+^, Co^2+^) connected by imidazolate ligands through coordination bonds, with different zeolite topological structures including SOD (ZIF-8) [58], GME (ZIF-69) [59], RHO (ZIF-71) [60], etc. Their tailorable porosity, high surface area, and excellent stability make them highly versatile for membrane separation, especially in gas separation. Furthermore, the milder syntheses compared to other MOF families allows for preparation under room temperature in large quantities. In this section, a detailed introduction to the fabrication strategies of ZIF membranes is provided, including in situ solvothermal synthesis, secondary growth, electrochemical deposition, counter diffusion growth, liquid phase epitaxy, and solvent-free synthesis.

### 3.1. In Situ Solvothermal Synthesis

**Figure 2:**
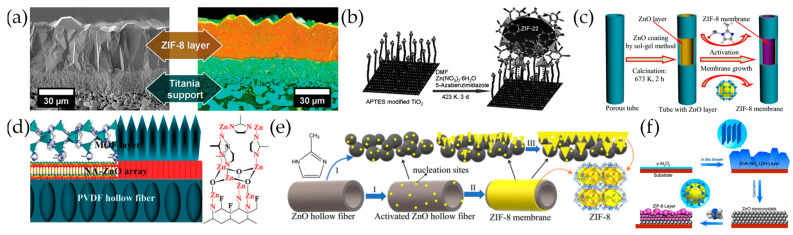
(**a**) (left) SEM image of the cross section of a simply broken ZIF-8 membrane. (right) EDXS mapping of the sawn and polished ZIF-8 membrane (color code: orange, Zn; cyan, Ti). Reprinted with permission from Ref. [61]. Copyright American Chemical Society, 2009. (**b**) Preparation of a ZIF-22 membrane by using APTES as covalent linker between the ZIF-22 membrane and the titania support. Reproduced with permission from Ref. [62]; published by John Wiley and Sons, 2010. (**c**) ZIF-8 membrane preparation procedure of modifying hollow fiber *α*-Al_2_O_3_ tubes with a ZnO sol. Reproduced with permission from Ref. [69]; published by Royal Society of Chemistry, 2013. (**d**) Scheme of the chemical structure and morphology of the prepared. Reproduced with permission from Ref. [71]; published by THE SOCIETY, 2014. (**e**) Proposed mechanism for the preparation of ZIF-8 membrane on ZnO hollow fiber. Reprinted with permission from Ref. [73]. Copyright American Chemical Society, 2020. (**f**) Fabrication of an ultrathin ZIF-8 membrane by partial conversion of a nanosized ZnO buffer layer derived from the ZnAl-NO_3_ LDH precursor membrane. Reproduced with permission from Ref. [74]; published by John Wiley and Sons, 2015.

In 2009, Bux et al. [61] employed microwave-assisted solvothermal synthesis to fabricate a crack-free, dense polycrystalline ZIF-8 layer on a porous titanium dioxide substrate, which exhibited a separation factor of 11.2 for a H_2_/CH_4_ mixture (1:1), marking the first application of ZIF materials on MOF membranes (Figure 2a). To increase the nucleation sites, the substrate was modified by 3-aminopropyltriethoxysilane (APTES), the amino group which can coordinate with metal ions or undergo a condensation reaction with ligand groups in the solution, thereby promoting the growth of continuous MOF membranes and strengthening the connection between MOFs and substrates. Utilizing APTES-modified substrates to facilitate nucleation, various well-intergrown and defect-free ZIF membranes have been attained, including ZIF-22 [62], ZIF-95 [63], ZIF-8 [64], and ZIF-9 [65], achieving excellent gas separation factors (Figure 2b). In addition to APTES, polydopamine is another commonly used organic ligand in the preparation of various ZIF membranes, such as ZIF-8 [66], ZIF-9 [67], ZIF-90 [67], ZIF-100 [68], etc.

In addition to organic modification, using ZnO(-modified) substrates can generate a large number of nucleation sites by interacting with ligands in the synthesis solution. Zhang et al. [69] modified hollow fiber *α*-Al_2_O_3_ tubes with a ZnO solution, which was subsequently treated with a 2-methylimidazole (2-mIm) solution to create uniform nucleation sites where a symbiotic, defect-free ZIF-8 membrane was in situ synthesized on the prepared substrate, with an ideal permeation selectivity of 8.2 and 8.1 for H_2_/N_2_ and H_2_/CH_4_, respectively (Figure 2c). The group further used a ZnO nanorod-modified porous ceramic substrate to induce the uniform nucleation of ZIF-8 [70]. Similarly, Li et al. [71] grew nanoscale thick NA-ZnO sheets on the surface of PVDF hollow fibers for the in situ solvothermal synthesis of ZIF-7 membranes (Figure 2d). Kong et al. [37] deposited a ZnO layer on the macroporous Al_2_O_3_ tube through manual rubbing, achieving a highly stable ZIF-8 membrane. Hara et al. [72] formed a ZnO layer by calcining porous *α*-alumina hollow capillary supports which were pre-immersed in a Zn(NO_3_)_2_ aqueous solution, for the in situ solvothermal synthesis of ZIF-8 films. Wang et al. [73] directly used ZnO hollow fibers as a substrate for the in situ solvothermal synthesis of ZIF-8 membranes (Figure 2e).

Using ZnO-modified substrates can allow for the in situ formation of ZIF membranes within a ligand solution in the absence of homogenously dissolved metal precursors. By the controlled calcination of a ZnAl-NO_3_ layered double hydroxide membrane, Liu et al. [74] fabricated a ZnO buffer layer on porous *γ*-Al_2_O_3_ substrates by the controlled calcination of a ZnAl-NO_3_ layered double hydroxide (LDH) membrane, which was subsequently thermally treated with a 2-mIm solution in H_2_O/DMF (DMF = N,N-dimethylformamide), achieving a 160 nm thick ZIF-8 membrane with an H_2_/CH_4_ (1:1) separation factor of 83.1 and H_2_ permeance of 1.4 × 10^–8^ mol·m^–2^·s^–1^·Pa^–1^ (Figure 2f). Through the ALD method, Ji et al. [75] deposited a 2 nm thick ZnO layer on the porous *α*-Al_2_O_3_ substrate, which was then transformed into a 180 nm thick, well-symbiotic ZIF-8 membrane in the 2-mIm solution. Li et al. [76] used Zn(OH)_2_ instead of ZnO as a metal source, where the ZIF-8 membrane with a thickness of 2.5 μm was fabricated on anodic aluminum oxide (AAO). The group further demonstrated the optimal temperature range from –2.5 to 2.5 °C for the ZIF-8 membrane in situ solvothermal synthesis, allowing for precise control over the nucleation and growth rates of ZIF-8, resulting in a separation factor for C_3_H_6_/C_3_H_8_ (1:1) of about 190 and a C_3_H_6_ permeance of 2.9 × 10^–8^ mol·m^–2^·s^–1^·Pa^–1^ [77].

Wang et al. [78] developed a ZIF-62 MOF glass membrane which was fabricated using a melt-quenching treatment of an in situ solvothermally synthesized polycrystalline ZIF-62 MOF membrane on a porous ceramic alumina support. The molten ZIF-62 phase penetrated into the nanopores of the support and eliminated the formation of intercrystalline defects in the resultant glass membrane. The molecular sieving ability of the MOF membrane is remarkably enhanced via vitrification. The separation factors of the MOF glass membrane for H_2_/CH_4_, CO_2_/N_2_, and CO_2_/CH_4_ mixtures are 50.7, 34.5, and 36.6, respectively. In addition, self-supported ZIF-62 glass membranes were prepared by a polymer-thermal-decomposition-assisted melting strategy, showing a CH_4_ permeance of 30,000–50,000 GPU [79].

### 3.2. Secondary Growth

**Figure 3:**
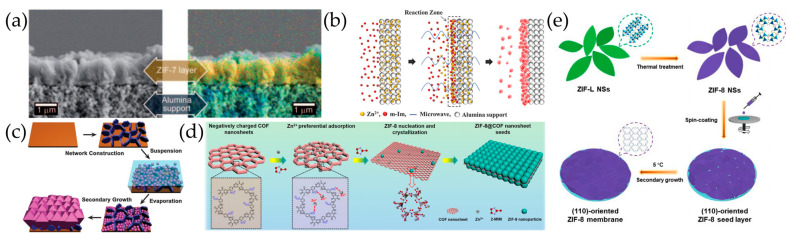
(**a**) EDXS mapping of the ZIF-7 membrane; orange Zn, cyan Al. Reproduced with permission from Ref. [80]; published by John Wiley and Sons, 2010. (**b**) Schematic illustration of rapid microwave-assisted seeding process. Reproduced with permission from Ref. [58]; published by THE SOCIETY, 2013. (**c**) Schematic illustration of the ZIF-8 membrane synthesis in the confined space of a vertically aligned LDH network illustrating the role of the LDH network: a ‘‘perch’’ for the seeds. Reproduced with permission from Ref. [87]; published by THE SOCIETY, 2014. (**d**) Schematic for the formation of ZIF-8@COF nanosheet seeds. Reproduced with permission from Ref. [91]; published by John Wiley and Sons, 2023. (**e**) Schematic illustration of preparing highly (110)-oriented ZIF-8 membrane. Reproduced with permission from Ref. [92]; published by Elsevier, 2022.

Pioneering work on the secondary growth of ZIF membranes has been reported by Li et al. [80], where the seed crystal layer was dip-coated on alumina substrates followed by the secondary growth using a microwave dielectric heating technique to achieve thin ZIF-7 membranes of 1.5 μm (Figure 3a). The H_2_ permeance of the membrane reached a plateau value of approximately to 8 × 10^–8^ mol·m^–2^·s^–1^·Pa^–1^ and the H_2_/N_2_ separation factor was 7.7.

Other seeding methods have also been investigated for the secondary growth of ZIF membranes including manual rubbing, reactive seeding, etc. By manual rubbing, Venna et al. [81] fabricated a ZIF-8 seed layer on *α*-Al_2_O_3_ porous support which further grew into 5–9 µm thick ZIF-8 membranes with CO_2_ permeances of 2.4 × 10^–5^ mol·m^–2^·s^–1^·Pa^–1^ and a CO_2_/CH_4_ separation selectivity of 4~7. Dong et al. [82] seeded the porous ZnO substrate through a reactive seeding route involving solvothermally treating the ZnO substrate in the ligand solution, followed by the secondary growth of the ZIF-78 membrane, which exhibited a H_2_/CO_2_ separation factor of 9.5. Kasik et al. [83] achieved continuous, highly crystalline, largely *c*-oriented ZIF-68 membranes on macroporous ZnO supports with a thickness of 50 μm by using a similar reactive seeding method.

To rapidly fabricate a ZIF-8 seed layer with a high packing density, Kwona et al. [58] subjected the *α*-Al_2_O_3_ support to microwave irradiation in a ligand solution following the treatment in a metal precursor solution (Figure 3b). The ZIF-8 membrane grown from this method exhibited a propylene/propane selectivity of up to 40. To effectively ‘‘root’’ the synthesis ingredients into the substrate, Li et al. [84] immersed the alumina support in melting 2-mIm followed by heat treatment in a zinc nitrate aqueous solution, where a dense and mechanically stable ZIF-8 layer with a thickness of about 12 μm was achieved on the treated support by secondary growth. To provide better-distributed nucleation sites, Fan et al. [85] utilized the nano-sized amorphous ZIF-78 instead of crystallized seeds to synthesize a well-intergrown ZIF-78 membrane on the porous silica substrate for the efficient pervaporative separation of cyclohexanone/cyclohexanol.

Similar to in situ solvothermal synthesis, substrate modification has also been demonstrated to be effective in enhancing heterogeneous nucleation in the secondary growth of ZIF-8 membranes. Huang et al. [86] used APTES-modified ceramic hollow fibers for seed fabrication and the membrane secondary growth of ZIF-8 on their inner surfaces. Liu et al. [87] constructed a network of crystallographically vertically aligned MgAl-CO_3_ LDHs walls on the porous *γ*-Al_2_O_3_ substrate to effectively collect seeds in a “perch” and prevent ZIF-8 seeds from peeling off during secondary growth (Figure 3c).

Secondary seeded growth has also been practiced on the tubular and meshed stainless steel supports where compact and mechanically stable ZIF-8 membranes have been accessed [88]; these membranes achieved outstanding separation efficiency for oil–water mixtures [89,90]. Recently, covalent organic frameworks (COFs) have also been used as substrates for the growth of ZIF membranes. Pu et al. [91] employed charged TpPa-SO_3_H COF nanosheets to efficiently induce the heterogeneous nucleation and epitaxy growth of ZIF-8 (Figure 3d). The evenly distributed –SO_3_^−^ groups on the COF nanosheet surface triggered long-range strong electrostatic attractions with Zn^2+^, leading to the preferential adsorption of Zn^2+^ onto the COF surface. The subsequent reaction between Zn^2+^ and 2-mIm led to the formation of ZIF-8@COF nanosheet seeds with highly-intergrown crystal grains. The subsequent secondary growth acquired ZIF-8 membranes with a thickness down to 100 nm, which exhibited a superior C_3_H_6_/C_3_H_8_ separation factor of over 90 with a C_3_H_6_ permeance of 600 GPU.

Compared to in situ solvothermal growth, secondary growth allows the accessibility of oriented membranes. Using the sub-micrometer sized ZIF-69 crystals seeds, Liu et al. [59] successfully synthesized highly *c*-oriented and well-intergrown ZIF-69 membranes on porous *α*-Al_2_O_3_ substrates, which exhibited enhanced selectivity and permeance for the CO_2_/CO mixture in comparison to polycrystalline ZIF-69 membranes by in situ crystallization. Furthermore, Zhang et al. [92] accessed a highly (110)-oriented ZIF-8 membrane by uniformly spin-coating (110)-oriented ZIF-8 nanosheet seeds on *α*-Al_2_O_3_ substrates, followed by a controlled in-plane secondary growth at a low temperature (Figure 3e).

### 3.3. Electrochemical Deposition

**Figure 4:**
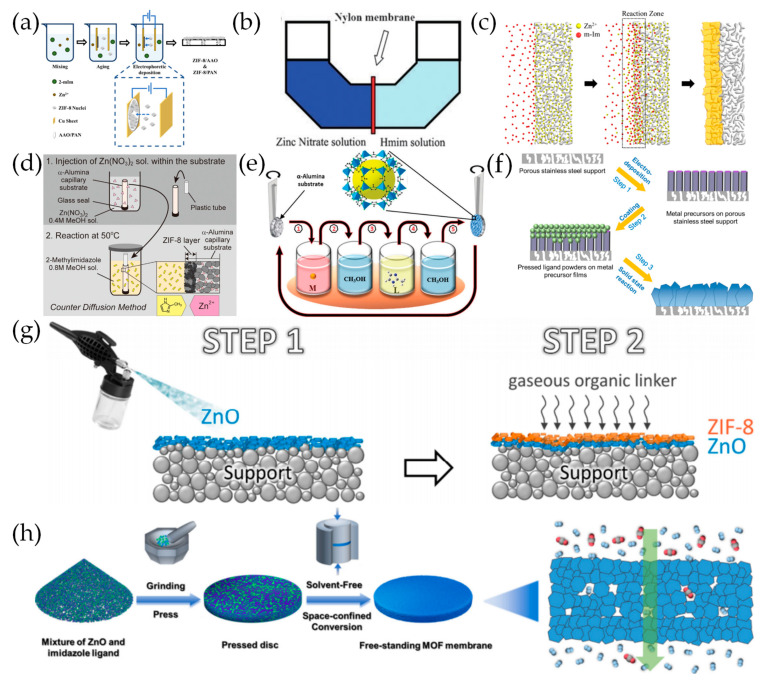
(**a**) Schematic diagram of the preparation process of ZIF-8 membrane with electrophoretic deposition method. Reproduced with permission from Ref. [94]; published by MDPI, 2022. (**b**) Diffusion cell for ZIF-8 film preparation. Reproduced with permission from Ref. [47]; published by THE SOCIETY, 2011. (**c**) Schematic illustration of the synthesis of ZIF-8 membranes using the counter diffusion-based in situ method. Reprinted with permission from Ref. [95]. Copyright American Chemical Society, 2013. (**d**) Schematic illustration of the synthesis of ZIF-8 membranes on porous *α*-alumina capillary substrate by counter diffusion method. Reproduced with permission from Ref. [97]; published by Elsevier, 2014. (**e**) A schematic representation of the LPE method used for the growth of the ZIF-8 membrane. Reproduced with permission from Ref. [99]; published by THE SOCIETY, 2014. (**f**) Schematic illustration of the synthesis of ZIF-8, ZIF-67 and ZIF-9 membranes through solid-state thermal conversion. Reproduced with permission from Ref. [55]; published by John Wiley and Sons, 2018. (**g**) Fabrication of ZIF-8 membranes by the two-step process. Step 1: spray coating of a ZnO layer. Step 2: vapor–solid reaction of gaseous 2-mIm and ZnO to ZIF-8. Reproduced with permission from Ref. [54]; published by Elsevier, 2019. (**h**) Schematic illustration of the SFSC approach for fabrication of free-standing MOF membrane. Reprinted with permission from Ref. [104]. Copyright American Chemical Society, 2023.

Zhu et al. [93] first successfully deposited thin ZIF-8 films onto porous stainless steel using an EPD method. Ji et al. [94] fabricated a ZIF-8 membrane on an unmodified AAO support using a one-step EPD process (Figure 4a). With a current of 0.65 mA/cm^2^ and deposition time of 60 min, a 300 nm thick ZIF-8 membrane was obtained, which exhibited a CO_2_ permeance of 334 GPU and a CO_2_/CH_4_ separation factor of 8.8, evidencing the defect-free structure.

### 3.4. Counter Diffusion Growth

Using a flexible polymer nylon substrate to separate zinc nitrate and a 2-mIm solution, Yao et al. [47] initially prepared ZIF-8 membranes with a thickness of 16 mm through counter diffusion growth at room temperature (Figure 4b). Based on the concept of counter diffusion, Kwon et al. [95] prepared a high-quality ZIF-8 membrane by first immersing the *α*-Al_2_O_3_ disk in a metal precursor solution for 1 h and then placing it in a ligand solution for 4 h at 120 °C, where the metal precursor and ligand solution diffused in opposite directions (Figure 4c). However, the rapid diffusion of the metal precursor and ligand solutions in alumina may lead to the formation of discontinuous films, which are susceptible to thermal structural degradation. Therefore, Janga et al. [96] deposited a *γ*-Al_2_O_3_ layer with a pore size of 5 nm on top of the *α*-Al_2_O_3_ disc to decrease the diffusion rates and as a protective layer to prevent thermal structural degradation to the ZIF-8 membranes.

Using the counter diffusion method, Hara et al. [97] prepared a 80 μm thick ZIF-8 membrane on a porous *α*-alumina capillary substrate, which was first immersed into the zinc nitrate solution with one end sealed, then transferred in a 2-mIm solution at 50 °C with two ends sealed (Figure 4d). Likewise, Huang et al. [60] prepared a ZIF-71 membrane on the outer surface of ceramic hollow fibers by vertically connecting an extended glass tube to the lumen of the substrate to increase the gravitational potential energy of the inner solution, making it easier for nutrients to pass through the defect of the membrane, which is conducive to the formation of a defect-free membrane.

### 3.5. Liquid Phase Epitaxy

Using the LPE method, Shekhah et al. [98] fabricated highly-oriented ZIF-8 thin films along the (110) direction with a controllable thickness that was proportional to the growth cycles at room temperature on an Au substrate functionalized with an –OH-terminated self-assembled monolayer (SAM). ZIF-8 membranes were also prepared on porous *α*-Al_2_O_3_ substrates by using a similar method (Figure 4e) [99]. Zvyagina et al. [100] assembled Zn-TCPP (TCPP = tetracarboxyphenyl porphyrin) surface-mounted metal–organic framework (SURMOF) films via the layer-by-layer coordination of zinc porphyrins with ZnAc_2_ clusters on the ultrathin surface coatings of graphene oxide (GO).

### 3.6. Solvent-Free Synthesis

By reacting the deposited ZnO layer with a melted 2-mIm solution at 433 K, Stassen et al. [56] first achieved the solvent-free synthesis of ZIF-8 thin films on silicon wafer and carbon steel supports. Furthermore, Hou et al. [55] electrodeposited a specific metal precursor (ZnO or Co(OH)_2_) layer on the porous stainless steel support and then coated the corresponding ligand powders on the top of the metal precursor, followed by a solid-state thermal conversion to form continuous defect-free ZIF-8, ZIF-67, and ZIF-9 membranes (Figure 4f).

Taking advantage of the volatility of an imidazole linker, Reif et al. [54] used a gaseous 2-mIm solution to conduct a gas–solid reaction with the ZnO layer sprayed on the substrates to generate defect-free ZIF-8 films (Figure 4g). Furthermore, a gel–vapor deposition (GVD) method was developed by Li et al. [101] where a Zn-based gel instead of ZnO was coated on ammoniated polyvinylidene fluoride (PVDF) hollow fibers and converted into an ultra-thin ZIF-8 film with a thickness of 17 nm by the vapor deposition of a 2-mIm solution, which exhibited an H_2_ permeance of up to 215.4 × 10^−7^ mol·m^–2^·s^–1^·Pa^–1^ with the H_2_/C_3_H_8_, CO_2_/C_3_H_8_, and C_3_H_6_/C_3_H_8_ (1:1) selectivity as high as 3400, 1030, and 70, respectively. Ma et al. [102] used ALD to deposit ZnO in an *α*-Al_2_O_3_ substrate coated with a about 5 mm *γ*-Al_2_O_3_ mesoporous layer followed by a 2-mIm vapor treatment. Subsequently, it was partially transformed to a ZIF-8 membrane which showed a stable separation factor for C_3_H_6_/C_3_H_8_ of about 50 to 70 and a C_3_H_6_ flux of 0.01 to 0.06 mol·m^–2^·s^–1^·Pa^–1^. Instead of using a ZnO layer, Bo et al. [103] used highly porous oxide fractal nanoparticle networks (FNNs) as a precursor to reacting with a 2-mIm vapor, the high porosity of which allows a full conversion into 23 µm thick ZIF-8 membranes. Recently, Gao et al. [104] adopted a new solvent-free space confined conversion (SFSC) approach to fabricate a series of free-standing MOF membranes including ZIF-8, Zn(EtIm)_2_, and Zn_2_(BIm)_4_, which involved sequentially grinding, pressing, and heating the physical mixture of ZnO and ligands (Figure 4h). The Zn(EtIm)_2_ membrane exhibited a high H_2_/CO_2_ selectivity of 22.1 with an excellent H_2_ permeance of 6268.7 GPU.

## 4. Fabrication of Cu-Based MOF Membranes

Cu-MOFs are mainly formed by the coordination of Cu^2+^ and carboxylic acid ligands [105], with the most extensively studied Cu-MOF being copper (II)-benzene-1,3,5-tricarboxylate [HKUST-1 or Cu_3_(BTC)_2_], which is comprised of Cu_2_(H_2_O)_2_ dimer units linked by benzene-1,3,5-tricarboxylate [106]. Its robust three-dimensional structure with large open pores, a high surface area, and abundant active metal sites, endows it with exceptional performance for various applications. Accordingly, the fabrication of Cu-MOF membranes have been attempted through in situ solvothermal synthesis, secondary growth, electrochemical deposition, liquid phase epitaxy, and solvent-free synthesis.

### 4.1. In Situ Solvothermal Synthesis

**Figure 5:**
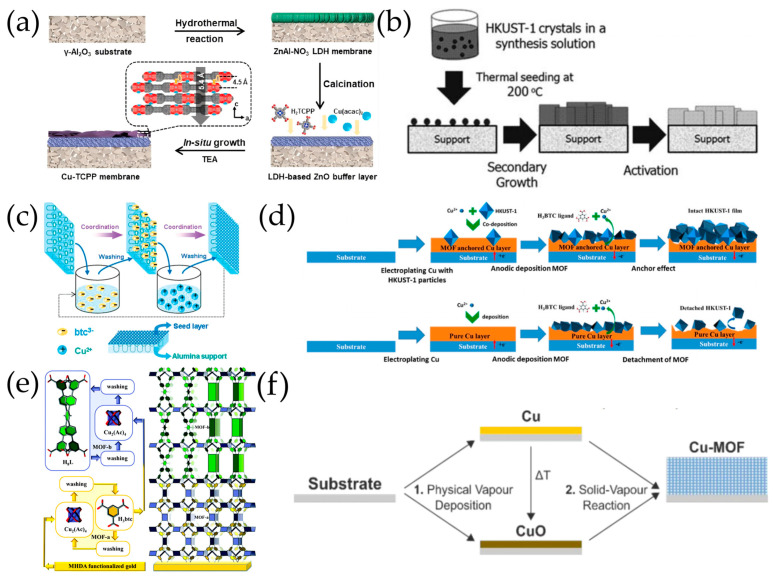
(**a**) Schematic illustration of preparing the well-intergrown, highly c-oriented ultrathin 2D Cu-TCPP membrane by in situ solvothermal growth. Reproduced with permission from Ref. [108]; published by Elsevier, 2021. (**b**) Schematic illustration of the synthesis procedure of HKUST-1 membranes by the thermal seeding and the secondary growth methods. Reproduced with permission from Ref. [40]; published by Royal Society of Chemistry, 2010. (**c**) Schematic diagram of SBS deposition of btc^3–^ and Cu^2+^ on alumina support. Reprinted with permission from Ref. [111]. Copyright American Chemical Society, 2011. (**d**) Schematic illustration of the anodic electrodeposition of HKUST-1 film. Reproduced with permission from Ref. [116]; published by Elsevier, 2023. (**e**) Schematic illustration of the LPE synthesis of MOF-on-MOF thin film on MHDA-modified gold substrate. Reproduced with permission from Ref. [52]; published by Royal Society of Chemistry, 2017. (**f**) Chemical vapor deposition of Cu-based MOF thin films. Reproduced with permission from Ref. [124]; published by Royal Society of Chemistry, 2019.

Guo et al. [107] achieved the in situ solvothermal synthesis of a Cu_3_(BTC)_2_ membrane supported by a copper net, which also acted as a metal source in addition to the Cu^2+^ ions in the reaction solution. The in situ solvothermal synthesis of another Cu-MOF membrane, 2D Cu-TCPP (TCPP = tetrakis (4-carboxyphenyl) porphyrin), was achieved on porous *α*-Al_2_O_3_ substrates by Song et al. [108]. Substrate surface decoration with LDH-based ZnO buffer layers, employing Cu(acac)_2_ as the copper source and triethylamine (TEA) additives in the precursor solutions were found to be crucial in preparing 2D Cu-TCPP membranes with the desired microstructure (Figure 5a). The resulting 2D Cu-TCPP membranes were well-intergrown and highly *c*-oriented with a thickness of 80 nm.

### 4.2. Secondary Growth

Pioneering work on the secondary growth fabrication of HKUST-1 membranes has been conducted by the Gascon group [109] on *α*-Al_2_O_3_ supports, which were spin-coated by a Cu_3_(BTC)_2_ coordination polymer. Various inoculation methods have been investigated for Cu-MOF seeding, including thermal seeding, step-by-step (SBS) seeding, the layer-by-layer method, reactive seeding, etc. Thermal seeding has been conducted by dropping a dispersion of HKUST-1 crystals containing organic ligands and copper species on the surface of hot *α*-Al_2_O_3_ supports for the secondary growth of continuous HKUST-1 membranes (Figure 5b) [40]. Noh et al. [110] used the thermal spray seeding method which combined thermal seeding and pressurized spraying to produce uniformly distributed HKUST-1 seed crystals strongly bonded to an *α*-Al_2_O_3_ support for the secondary growth of a continuous crack-free HKUST-1 membrane. To fabricate a uniform seed layer, Nan et al. [111] developed a SBS seeding technique which involved sequentially dipping the polished side of the *α*-Al_2_O_3_ substrate in an H_3_BTC solution for 40 min, ethanol for 5 min, Cu(CH_3_COO)_2_ solution for 20 min, and ethanol for 5 min (Figure 5c). Likewise, Wang et al. [112] established an LBL seeding method which involved repeatedly soaking the APTES-modified porous Al_2_O_3_ substrate into the solutions of H_2_OEt-ipa and Cu(NO)_2_ to construct the seed layer for the secondary growth of a continuous and defect-free Cu(OEt-ipa) (OEt-ipa = 5-ethoxyisophthalate) membrane. Mao et al. [113] prepared HKUST-1 seeds on AAO substrates by reacting solid copper hydroxide nanostrand (CHN) thin films with H_3_BTC at room temperature, which were then directly used for the secondary growth of the HKUST-1 membranes. An example of a special inoculation method is the method used by Chen et al. [114]; these researchers facilely constructed a defect-free Cu-TCPP lamellar membrane using a triple-needle electrostatic atomization technology. Specifically, one needle atomizes small-sized Cu-TCPP MOF nanosheets to build a lamellar skeleton on a nylon support while in the meantime, the other two needles atomize Cu^2+^ and TCPP to secondarily assemble crystals among nanosheets. Ranjan et al. [35] prepared preferentially oriented and well-intergrown films of a microporous metal organic framework, which was built upon a copper paddlewheel and a V-shaped dicarboxylate ligand, as a result of the competitive grain growth of the randomly oriented seed layer on porous *α*-Al_2_O_3_ discs.

### 4.3. Electrochemical Deposition

Using anodic deposition, Ameloot et al. [44] prepared HKUST-1 films on copper electrodes and the thickness varied by simply altering the conditions. A higher voltage provides a higher concentration of metal ions near the surface and yields coatings of smaller crystals, while diluting the synthesis mixture with water could slow down crystal formation and yield larger crystals. Similarly, Voorde et al. [115] prepared Cu(CHDA), Cu(INA)_2_, and HKUST-1 (CHDA = trans-cyclohexane-1,4-dicarboxylate; INA = isonicotinate) films on pure copper electrodes. Anodic deposition is driven by the anodic dissolution of a metal substrate, which weakens the MOF-substrate adhesion and leads to the detachment of the MOF films, limiting the thickness of the MOF films that can be deposited with this technique. To grow thicker MOF films by anodic deposition, Guo et al. [116] proposed a strategy to improve the adhesion of MOF films by pre-depositing MOF particles into the substrate as “anchors”, by which HKUST-1 films with an average thickness of 42 μm were grown on a copper wafer (Figure 5d).

Xie et al. [117] proposed a hydrogen peroxide-assisted cathodic deposition (HPACD), where hydrogen peroxide is reduced to superoxide anions that trigger the deprotonation of MOF ligands. This method prevents the co-deposition of metal, and various pure MOF films were prepared via this approach, including HKUST-1, MIL-53(Fe), and MOF-5.

### 4.4. Liquid Phase Epitaxy

On a COOH-terminated SAM surface, Shekhah et al. [118] first used a step-by-step route to prepare HKUST-1 films by repeated immersion cycles, first in a solution of a metal precursor and subsequently in a solution of organic ligands. The step-by-step deposition of the multilayer was monitored for the first time through surface plasmon resonance with a submonolayer resolution, yielding homogeneous, highly crystalline HKUST-1 films. Bétard et al. [119] prepared pillared layered [Cu_2_L_2_P]_n_ (L = dicarboxylate linker, P = pillaring ligand) MOF membranes on porous TiO_2_ and *α*-Al_2_O_3_ discs by stepwise liquid-phase deposition, where the substrates were sequentially treated by a copper precursor solution, ethanol, dicarboxylate linker solution, ethanol, pillaring ligand solution, and ethanol for 90 cycles. Using the LPE method, Chang et al. [120] grew enantiopure [Cu_2_(cam)_2_dabco]_n_ (H_2_cam: camphoric acid; dabco: 1,4-diazabicyclo [2.2.2]octane) thin films with a preferred [110] orientation on carboxyl-containing N-heterocyclic carbene self-assembled monolayer-modified gold [Au(NHC)] substrates for sensing applications. Continuous and dense NOTT-100 ([Cu_2_(bptc)(H_2_O)_2_]; bptc = biphenyl-3,3′,5,5′-tetracarboxylate) and NOTT-101 ([Cu_2_(tptc)(H_2_O)_2_]; tptc = [1,1′:4′,1”]terphenyl-3,3″,5,5″-tetracarboxylate) films were also prepared on surface acoustic wave sensors using the LBL method, which respond reversibly to vapors of water, acetone, and n-hexane [121].

In view of the capability of the LPE method to precisely control film thickness and orientation during the growth process, Chernikova et al. [52] fabricated the MOF-on-MOF heterostructured thin films by the sequential deposition of two different MOFs layers on a COOH-terminated gold substrate (Figure 5e). An initial HKUST-1 layer was deposited by liquid phase epitaxial growth from H_3_BTC and the copper paddlewheel. The [001] directional growth allowed the successful epitaxial growth of the second MOF, Cu-**tbo**-MOF-5, due to the perfect lattice match along this direction. Ikigak et al. [122] further precisely aligned three different MOF layers using the LPE method, which involved the first Cu_2_(BPDC)_2_ layer (BPDC = biphenyl-4,4′-dicarboxylate) grown on an oriented Cu(OH)_2_ film, a second aligned layer [Cu_2_(BDC)_2_ (BDC = benzene 1,4-dicarboxylate), or Cu_2_(BPYDC)_2_, (BPYDC = 2,2′-bipyridine-5,5′-dicarboxylate)] and a top layer of Cu_2_(NDC)_2_ (NDC = naphthalene 2,6-dicarboxylate). Each MOF layer was heteroepitaxially matched and aligned to the Cu(OH)_2_ substrate in all three crystallographic directions.

Wang et al. [123] systematically explored the influence of water on the stepwise layer-by-layer LPE growth of Cu paddlewheel-based MOFs through mixing various amounts of water in linker solutions. Integrating 5% (of volume) of water in the linker solution is favorable for the fabrication of Cu_3_(BTC)_2_ and CuBDC SURMOFs with high crystallinity, preferred orientation, homogeneous texture, and enhanced sorption capacity.

### 4.5. Solvent-Free Synthesis

Ahvenniemi et al. [125] demonstrated that crystalline thin films of copper (II) terephthalate (Cu-TPA) with a paddlewheel MOF-2 type structure can be deposited in an atomic/molecular layer-by-layer manner using two gaseous precursors on p-type Si (100) substrates. Recently, Gikonyo et al. [126] expanded the scope of the ALD/MLD layer-by-layer growth of crystalline MOF films and demonstrated the facile fabrication of 3D Cu-TPA films on different kinds of amorphous (Si with native oxide) or crystalline (sapphire, FTO) inorganic substrates. They also demonstrated a heteroepitaxial growth achievable in the vapor phase by using DMOF-1 (Zn-TPA-DABCO) single crystals as the starting surface with a lattice matching topology. Stassin et al. [124] reported the out-of-plane growth of CuBDC and CuCDC films via chemical vapor deposition (CVD) where the Cu or CuO layer reacted with vaporized 1,4-benzendicarboxylic acid (H_2_BDC) and trans-1,4-cyclohexanedicarboxylic acid (H_2_CDC) linkers (Figure 5f).

## 5. Fabrication of Zr-Based MOF Membranes

As one of most important MOF families, Zr-based MOFs are primarily built from the hexanuclear cluster of tetravalent zirconium with multitopic carboxylate ligands, which attracted great interest for various applications due to their high porosity, structural robustness, and network versatility. Accordingly, the fabrication of Zr-based MOF membranes for membrane separation purposes has been an intriguing area of research. Despite of the versatility of Zr-based MOF skeletons with various building blocks and geometries, research on pure Zr-based MOF membranes have primarily focused on the first reported UiO-66 and its isostructural derivatives, which are constructed from the 12-connected hexanuclear zirconium cluster (Zr_6_) and terephthalate linkers. The aforementioned MOF membrane fabrication techniques that have been employed for ZIFs and Cu-MOFs have also been implemented for the fabrication of Zr-based MOF membranes, such as in situ solvothermal fabrication, secondary growth, electrochemical deposition, counter diffusion growth, solvent-free synthesis, and liquid phase epitaxy.

### 5.1. In Situ Solvothermal Synthesis

**Figure 6:**
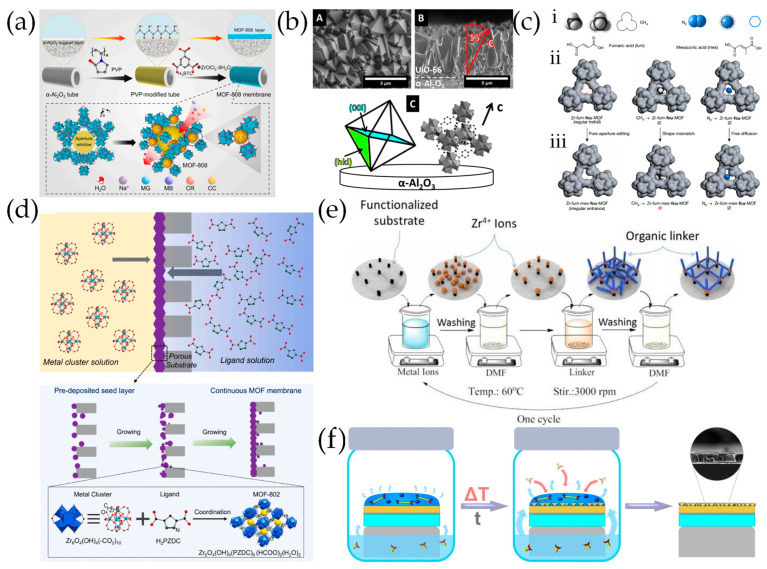
(**a**) Schematic diagram of the fabrication of polycrystalline MOF-808 membrane. Reproduced with permission from Ref. [131]; published by Elsevier, 2023. (**b**) Exemplary SEM images of the supported UiO-66 membrane in (A) top view and (B) cross section. (C) Schematic representation of the octahedrons orientation within the UiO-66 layer and their orientation to the support surface. Reprinted with permission from Ref. [134]. Copyright American Chemical Society, 2017. (**c**) Schematic illustrations of pore-aperture editing and shape mismatch-induced separation based on shape difference. (i) Molecular configurations of CH_4_ and N_2_, and structures of fumaric acid and mesaconic acid. (ii) Illustrations of the regular trefoil-shaped pore aperture of Zr-*fum*-**fcu**-MOF and the free diffusions of both CH_4_ and N_2_ molecules. (iii) Illustrations of the irregular entrance of Zr-*fum*-*mes*-**fcu**-MOF created by subtle pore-aperture editing. Reproduced with permission from Ref. [27]; published by Springer Nature, 2022. (**d**) Scheme depicting the crystal seeds induced interfacial growth approach for fabricating MOF-802 membrane. Reproduced with permission from Ref. [139]; published by Elsevier, 2024. (**e**) Schematic diagram for the synthesis of UiO-66-NH_2_ SURMOF through LPE. Reproduced with permission from Ref. [142]; published by John Wiley and Sons, 2020. (**f**) Schematic representation of the vapor-assisted conversion process for the fabrication of oriented MOF films. Reprinted with permission from Ref. [143]. Copyright American Chemical Society, 2018.

Liu et al. [34] first fabricated pure UiO-66 polycrystalline membranes on alumina hollow fibers using an in situ solvothermal synthesis, which exhibited an excellent multivalent ion rejection for Ca^2+^, Mg^2+^, and Al^3+^ of 86.3%, 98.0%, and 99.3% with a moderate permeance of 0.14 L·m^−2^·h^−1^·bar^−1^. Liu et al. [127] further prepared UiO-66 polycrystalline films on the yttria-stabilized zirconia hollow fibers (YSZ HF), which could chemically bond with BDC ligands and promote the heterogeneous nucleation of UiO-66. The as-prepared UiO-66 membrane exhibited ultra-high separation factors of over 45,000 for 10 wt% water/*i*-butanol, water/furfural, and water/tetrahydrofuran and a total flux of up to approximately 6.0 kg·m^−2^·h^−1^. To enhance the adherence to the substrate, Wan et al. [128] further used APTES-modified Al_2_O_3_ tubes to synthesize well-intergrown UiO-66-NH_2_ membranes, which showed high desalination performances and good stability. Decorating the *α*-Al_2_O_3_ substrate with ZrO_2_, including ZrO_2_ sol [129] and ZrO_2_ powder [130], has also been attempted to introduce more nucleation and anchoring sites for the solvothermal growth of continuous UiO-66-NH_2_ membranes. Wu et al. [131] used polyvinylpyrrolidone (PVP) to modify the porous *α*-Al_2_O_3_ substrate to prepare a well-intergrown polycrystalline MOF-808 membrane (Figure 6a). Strong affinity interactions between polar groups –N^+^ = C–O^–^ in the PVP chain and Zr^4+^ ions in the MOF-808 framework facilitated heterogeneous nucleation. The separation factor of the membrane for salt/dye was as high as 287.3, and the permeance of water was 4.37 L·m^–2^·h^–1^·bar^–1^.

### 5.2. Secondary Growth

The addition of monocarboxylic acid as a synthetic modulator is often required for the secondary growth of Zr-based MOF membranes. This addition can promote the formation of larger crystal grains and the orientation-preferred growth of the membrane. Liu et al. [132] involved acetic acid as a regulator into the secondary growth of UiO-66-CH_3_ membranes on a porous Ni support sheet where a dense, intergrown membrane layer is formed by a 24 h growth period over a temperature range from 120 to 160 °C. Miyamoto et al. [133] added acetic acid and water into the repeated solvothermal synthesis process, resulting in the formation of a highly oriented UiO-66 film with a monocrystalline layer. It was explained that acetic acid favored the highly orientated film by regulating the crystal growth rate, and water can promote the intergrowth of UiO-66 crystals by strengthening the hydrolysis of Zr precursors. Furthermore, Friebe et al. [134] acquired (002)-oriented UiO-66 membranes on the *α*-Al_2_O_3_ support by adding benzoic acid into the process of secondary growth (Figure 6b). The UiO-66 coated mesh membrane was also attained by a room temperature secondary growth on the stainless steel mesh, which was ultrasonically pre-seeded within a UiO-66 seed nanocrystal suspension in deionized water [135]. The prepared membrane exhibited a remarkable separation efficiency of over 99.99% for the oil–water separation with a high water permeation flux of 12.7 × 10^4^ L·m^–2^·h^–1^.

### 5.3. Electrochemical Deposition

Pioneering work on the electrochemical deposition of Zr-based MOFs have been accomplished by Hod et al. [43] where UiO-66 and NU-1000 in nano- and micro-particulate, thin-film form have accomplished on fluorine-doped tin oxide (FTO) glasses using electrophoretic deposition. Subsequently, the anodic and cathodic deposition of UiO-66 films has been investigated by Stassen et al. [136]. In anodic deposition, adjusting the concentration of acetic acid can control not only the crystallite size but also the morphology of films. Increasing the concentration of acetic acid can shift the deposition process from mainly anodic deposition to cathodic deposition. Using a facile cathodic deposition method, Li et al. [137] prepared a Janus MOF membrane by separately depositing ZIF-8 and UiO-66(COOH)_2_ on two sides of an AAO substrate. The asymmetric structure and surface charge distribution enhance the ion charge separation and improve the energy-harvesting performance, resulting in an output power density of 3.44 W/m^2^ at a 1000-fold concentration gradient. In cathodic deposition of UiO-66 films, Xie et al. [138] added lithium nitrate to the deposition bath as a probase to accelerate the electrochemical deprotonation of the linkers, leading to fast, convenient, and controllable fabrication of continuous UiO-66 films on AAO. The membranes display excellent ion transport with a Li^+^/Mg^2+^ selectivity of 286 and permeance of 11.2 mol·m^–2^·h^–1^.

Through electrochemical methods, Zhou et al. [27] successfully deposited Zr-*fum*_(100 − x)_-*mes*_x_-***fcu***-MOF membranes on the Pt-coated porous support by adding different ratios of fumaric acid (*fum*) and mesaconic acid (*mes*) to the solution of Zr_6_ clusters. These membranes have specific shaped pore sizes that block the transport of tetrahedral methane while allowing for linear nitrogen permeation (Figure 6c). The Zr-*fum*_67_-*mes*_33_-***fcu***-MOF membrane with a 2:1 ratio of *fum* to *mes* provided the highest N_2_/CH_4_ selectivity of 15 and N_2_ permeance of 3057 GPU.

### 5.4. Counter Diffusion Growth

Zhang et al. [139] reported fabrication of MOF-802 membranes by a crystal-seed-induced interfacial growth approach. A MOF-802 seeding layer was deposited on the substrate surface, providing nucleation sites to induce the intergrowth of MOF-802 into crystalline membrane when metal source and ligand diffuse in opposite directions and coordinatively react on the surface of porous substrate. The resulting continuous MOF-802 membrane displays excellent H_2_/CO_2_ separation performance with H_2_ permeance of 3540 GPU and selectivity of 23.1 (Figure 6d).

### 5.5. Liquid Phase Epitaxy

Zr-based MOFs were constructed by multitopic ligands connecting Zr_6_ clusters which, theoretically, have 12 symmetric connection sites, commonly 8 ~ 12 connected in most Zr-based MOFs. The high coordination number presents a challenge when attempting to deposit Zr-based MOFs with a desired 3D periodic network of acceptable quality using the stepwise liquid phase epitaxy process. Semrau et al. [140] reported the LPE growth of highly porous and crystalline UiO-66 films by alternately immersing the native silicon dioxide substrates (UV activated) into the ethanol solution of metal source zirconium methacrylate clusters [Zr_6_O_4_(OH)_4_(OMc)_12_], the ethanol solution of H_2_BDC, and rinsing the substrate with ethanol between the two steps. Methacrylic acid (McOH) was added into both the metal source and linker solution as a coordinative modulator during the LPE process. Using a similar protocol, the group subsequently prepared UiO-66-NH_2_ membranes on silicon substrates with a native oxide surface (piranha acid-activated) [141].

Using hydroxyl-functionalized flat gold films supported on Si wafers, high-quality monolithic UiO-66-NH_2_ thin films were prepared by Hashem et al. [142] via a low temperature liquid phase epitaxy method (Figure 6e). Specifically, the substrates were alternately immersed in DMF solutions of ZrCl_4_ (in presence of HCl) and the 2-aminoterephthalic acid organic linker, followed by rinsing with a pure DMF solvent after each immersion step. The overall thickness of the deposited thin films could be adjusted by the number of growth cycles. The same method was also used to prepare a thin UiO-66-NH_2_ SURMOF layer on a filter paper substrate, which demonstrated excellent separation for a mixture of a methylene blue and methyl orange aqueous solution.

### 5.6. Solvent-Free Synthesis

Lausund et al. [144] first prepared UiO-66 thin film on Si(001) substrates in an all-gas-phase process with the aid of ALD. Sequential reactions of ZrCl_4_ and 1,4-benzenedicarboxylic acid produce amorphous organic–inorganic hybrid films that are subsequently crystallized to the UiO-66 structure by treatment in an acetic acid vapor. Virmani et al. [143] prepared UiO-66, UiO-66(NH_2_), UiO-67, and UiO-68(NH_2_) films on gold surfaces modified with thiol SAMs using the vapor-assisted conversion (VAC) method (Figure 6f). Upon exposure to DMF and the acetic acid vapor at an elevated temperature of 100 °C for 3 h, precursors containing ZrOCl_2_, dicarboxylic acid, and the modulator acetic acid in a cast solution layer on the substrate were converted into a porous continuous MOF film oriented along the (111) crystal axis. Recently, Luo et al. [145] deposited Zr_6_-based NU-906 on the porous SiO_2_ support by introducing a trace amount of formic acid vapor to induce the formation of continuous pinhole-free MOF thin films with a preferred orientation in the fashion of an in situ recrystallization, which exhibited stable high separation factors of 2630 and 501, and fluxes of 1.45 and 1.41 kg·m^−2^·h^−1^ for n-butanol/water and MeOH/MTBE, respectively.

## 6. Fabrication of Al-Based MOF Membranes

Strong Al-O bond endows excellent structural robustness and potentials in various areas. The investigations of pure Al-MOFs membranes have been conducted mainly on the CAU (Christian Albrechts University) series and the Al-based MIL (Materials of Institute Lavoisier) series, including CAU-10-H, CAU-1, MIL-53, MIL-120, etc. These Al-MOFs are constructed from distinct secondary building units of Al3+ and different organic ligands, with different geometry structure. The fabrication methods of these Al-MOF membranes mainly involve in situ solvothermal synthesis and secondary growth.

### 6.1. In Situ Solvothermal Synthesis

**Figure 7:**
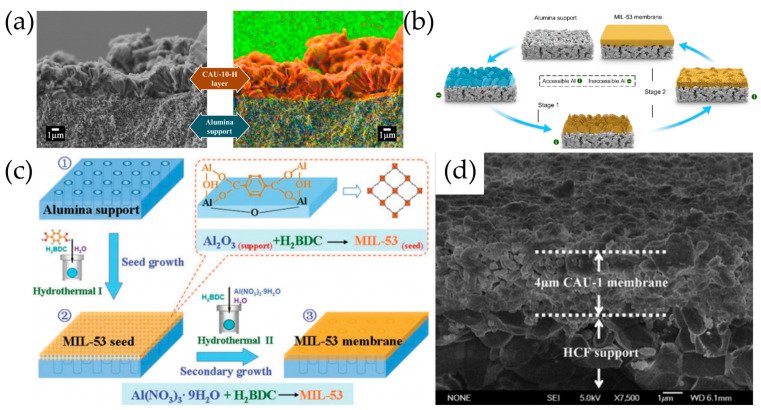
(**a**) Cross-sectional SEM image and the corresponding EDXS-mapping of the CAU-10-H membrane; orange–red C. Reproduced with permission from Ref. [146]; published by Elsevier, 2016. (**b**) Schematic of the LDH layer as a trigger for the growth of the MIL-53 membrane at stage 1, and a modulator for the accessibility of alumina support to BDCA solution at stage 2. Reproduced with permission from Ref. [149]; published by John Wiley and Sons, 2023. (**c**) Schematic diagram of preparation of the MIL-53 membrane on alumina support via the RS method. Reproduced with permission from Ref. [39]; published by Royal Society of Chemistry, 2011. (**d**) Cross-sectional view of the CAU-1 membrane. Reproduced with permission from Ref. [151]; published by Elsevier, 2013.

Using in situ solvothermal synthesis, Jin et al. [146] prepared a novel Al-MOF membrane of CAU-10-H on an *α*-Al_2_O_3_ disc, which was constructed from aluminum ions and the V-shaped linker molecule 1,3-benzene dicarboxylic acid (Figure 7a). This membrane, with a thickness of about 6 μm, showed mixed gas separation factors of 10.5 and 74.7 for H_2_/CO_2_ and H_2_/CH_4_ binary mixtures (1:1), with an H_2_ permeance of 1.53 × 10^–8^ mol·m^–2^·s^–1^·Pa^–1^. Liu et al. [147] successfully synthesized a novel heterogeneous MIL-121/118 membrane on the surface of macroporous glass frits using in situ solvothermal synthesis. The membrane exhibited permselectivity towards hydrogen, with separation factors of 10.7, 8.9, and 7.5 for H_2_/CO_2_, H_2_/CH_4_, and H_2_/N_2_ gas mixtures (1:1), respectively, and with an average H_2_ permeance of 7.83 × 10^–8^ mol·m^–2^·s^–1^·Pa^–1^. Continuous aluminum MOF-303 membranes were prepared on *α*-Al_2_O_3_ substrates via an in situ solvothermal synthesis method, exhibiting a high rejection of divalent ions (93.5% for MgCl_2_ and 96.0% for Na_2_SO_4_) and unprecedented water permeability (3.0 L·m^−2^·h^−1^·bar^−1^ μm) [148]. Wang et al. [149] utilized a continuous layer of ZnAl-CO_3_ LDH nanoflakes on an alumina support as a template, which was further chemically converted to a MIL-53 membrane upon immersion into the 1,4-benzenedicarboxylic acid (BDCA) solution (Figure 7b). The LDH template not only provides an Al source, but also dynamically regulates the availability of Al nutrients from the alumina support, resulting in a synergistic effect for producing membranes with a highly compact architecture. The MIL-53 membrane realized nearly complete dewatering from formic acid and acetic acid solutions and maintained stability in a continuous pervaporation of over 200 h.

### 6.2. Secondary Growth

By using the reactive seeding method that involved the reaction between the inorganic support and the organic precursor in a single stage, Hu et al. [39] prepared a uniform MIL-53 seed layer which was further used to synthesize a well-intergrown MIL-53 membrane with a thickness of 8 μm on porous alumina supports (Figure 7c). Zhang et al. [150] used colloidal seeds to form seed layers and then prepared NH_2_-MIL-53(Al) membranes with a thickness of 15 μm on macroporous glass frit discs. The NH_2_-MIL-53(Al) membrane showed selectivity for H_2_ with separation factors of 20.7, 23.9, and 30.9 at room temperature for H_2_ over CH_4_, N_2_, and CO_2_ (1:1), and an H_2_ permeance of 1.6 × 10^–7^ mol·m^–2^·s^–1^·Pa^–1^.

*α*-Al_2_O_3_ hollow ceramic fibers (HCFs) can play dual roles in the Al-based MOF membrane preparation, namely as an aluminum source and as a support. Zhou et al. [151] successfully synthesized high-quality CAU-1 membranes with a thickness of approximately 4 μm by secondary growth, assisted with homogenously deposited CAU-1 nanocrystals with a size of 500 nm as seeds (Figure 7d). Fan et al. [152] synthesized continuous and compact MIL-120 membranes supported by HCFs through the secondary growth of the seed layers formed through an in situ solvothermal process. To shorten the membrane fabrication time, Jin et al. [153] developed microwave-assisted secondary growth to accelerate the well-intergrown hydrophilic CAU-10-H membranes on *α*-Al_2_O_3_ discs, which exhibited a total flux of 493 g·m^–2^·h^–1^ for 90 wt% ethanol feed with a separation factor of 148 at 65 °C.

## 7. Fabrication of Ni-Based MOF Membranes

Investigations of pure Ni-MOF membranes have been conducted on KAUST-7, Ni-MOF-74, and 2D Ni_3_(HITP)_2_ (HITP = 2,3,6,7,10,11-hexaiminotriphenylenesemiquinonate), etc. The preparation methods of these Ni-MOF membranes include in situ solvothermal synthesis, secondary growth, and counter diffusion growth.

### 7.1. In Situ Solvothermal Synthesis

**Figure 8:**
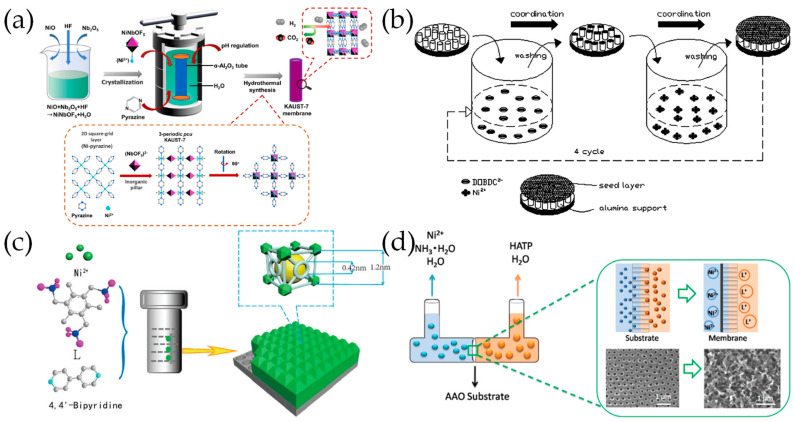
(**a**) Schematic diagram of the preparation process of KAUST-7 membranes by in situ synthesis assisted by fluorinated molecular building block. Reproduced with permission from Ref. [154]; published by Elsevier, 2022. (**b**) Schematic diagram of layer-by-layer method for the seeded samples. Reproduced with permission from Ref. [155]; published by Elsevier, 2012. (**c**) Schematic illustration of the fabrication of the [Ni_1.5_(4,4’-bipy)_1.5_H_3_L(H_2_O)_3_(H_2_O)_7_] membrane via the in situ seed-mediated secondary growth strategy. Reproduced with permission from Ref. [157]; published by Royal Society of Chemistry, 2017. (**d**) Schematic of preparation of 2D Ni_3_(HITP)_2_ MOF membranes by a contra-diffusion method (HATP = 2,3,6,7,10,11-hexaaminotriphenylene). Reproduced with permission from Ref. [49]; published by Royal Society of Chemistry, 2021.

The synthesis of KAUST-7 crystals requires the use of high concentrations of hydrofluoric acid (HF), which can corrode the *α*-Al_2_O_3_ support during the synthesis process of KAUST-7 membranes. Instead of corrosive HF, Lv et al. [154] included NiNbOF_5_ into the in situ solvothermal synthesis of a well-intergrown and defect-free tubular KAUST-7 membrane on coarse asymmetric *α*-Al_2_O_3_ tubes (Figure 8a). The pH manipulation of the synthesis solution proved to be crucial to the fabrication of defect-free KAUST-7 membranes and the optimal pH level was identified as 4.0. The thickness of the KAUST-7 membrane was about 10 μm, which exhibited an H_2_ permeance selectivity of 17.7 over CO_2_ with an H_2_ permeance of 2.2 × 10^–7^ mol·m^–2^·s^–1^·Pa^–1^ at 25 °C.

### 7.2. Secondary Growth

Lee et al. [155] used a layer-by-layer seeding technique followed by secondary growth crystallization to prepare continuous and defect-free Ni-MOF-74 membranes on an *α*-alumina support (Figure 8b). The secondary growth method has also been employed by Kang et al. [156] to fabricate new [Ni_2_(L-asp)_2_(pz)] (L-asp = L-aspartic acid, and pz = pyrazine) membranes with a thickness of 30~40 μm on nickel screens. The ultramicroporous nature of the membrane enabled high selectivity factors of 26.3, 17.1, and 38.7 for H_2_/CH_4_, H_2_/N_2_, and H_2_/CO_2_ at room temperature. Other than carboxylate connected Ni-MOFs, Yang et al. [157] construct a phosphonate-based MOF ([Ni_1.5_(4,4′-bipy)_1.5_H_3_L(H_2_O)_3_(H_2_O)_7_] (L = 2,4,6-trimethylbenzene-1,3,5-triyl)tris(methylene)triphosphonic acid) membrane on a porous anodic alumina membrane (PAAM) substrate using an in situ seed-mediated secondary growth strategy (Figure 8c). The thickness of the membrane reduced from about 450 μm to 200 μm as the synthesis temperature increased from 413 K to 453 K.

### 7.3. Counter Diffusion Growth

Jiang et al. [49] used a simple, effective, and environmentally friendly contra-diffusion method to produce an Ni_3_(HITP)_2_ MOF membrane on the interface of the ordered macroporous AAO support (Figure 8d). The as-prepared membrane with a thickness of about 600 nm is highly continuous and compact.

## 8. Fabrication of Ti-Based MOF Membranes

**Figure 9:**
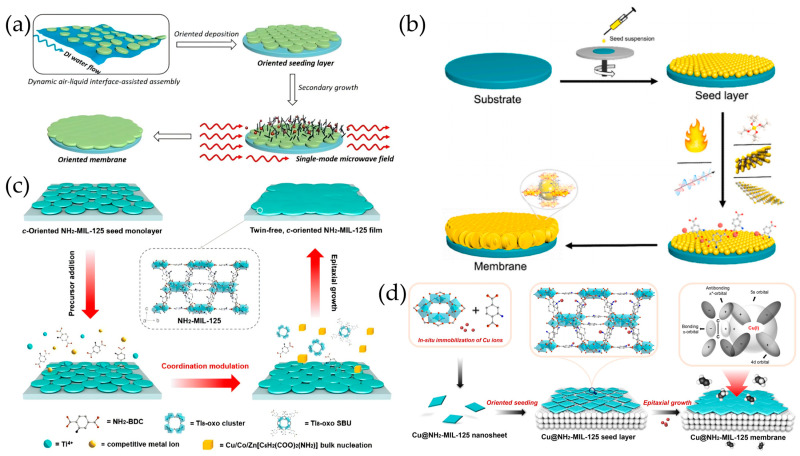
(**a**) Schematic illustration of the preparation procedure of highly *c*-oriented NH_2_-MIL-125(Ti) membrane by combining oriented seeding and controlled in-plane secondary growth (Red sphere: Ti^4+^ ion, black rod: NH_2_-BDC). Reproduced with permission from Ref. [158]; published by John Wiley and Sons, 2018. (**b**) Schematic diagram for solvothermal epitaxial growth of NH_2_-MIL-125 seed layer (red spheres: Ti^4+^ ions). Reproduced with permission from Ref. [159]; published by Elsevier, 2020. (**c**) Illustration of the preparation of twin-free and *c*-oriented NH_2_-MIL-125 film via competitive metal ion-based coordination modulation strategy by epitaxial growth. Reproduced with permission from Ref. [160]; published by Royal Society of Chemistry, 2022. (**d**) Scheme illustration of the preparation of highly *c*-oriented ultrathin π-complexation Cu@NH_2_-MIL-125 membrane. Reproduced with permission from Ref. [161]; published by John Wiley and Sons, 2023.

Research on Ti-based MOF membranes has primarily been conducted systematically by the Sun group, focusing on MIL-125(Ti), which is composed of titanium ions and terephthalic acid ligands, with a three-dimensional structure and large open pores. Initially, Sun et al. [158] developed a novel dynamic air–liquid interface-assisted self-assembly method for the deposition of closely packed and highly *c*-oriented NH_2_-MIL-125(Ti) seed monolayers on porous *α*-Al_2_O_3_ substrates, while the use of layered TiS_2_ as the metal source and the employment of single-mode microwave irradiation during epitaxial growth were found to be indispensable for maintaining the desired in-plane growth and suppressing the undesired twin growth (Figure 9a).

Subsequently, different Ti sources (titanium isopropoxide, TiS_2_, and MXene) and heating modes (conventional heating, multi-mode microwave heating, and single-mode microwave heating) were investigated by Sun et al. [159] for the preparation of NH_2_-MIL-125 membranes through secondary growth (Figure 9b). It was determined that NH_2_-MIL-125 membranes with TiS_2_ as the metal source under single-mode microwave heating exhibited the highest H_2_/CO_2_ selectivity (ca. 17.2) as well as considerable H_2_ permeance (1.7 × 10^–7^ mol·m^–2^·s^–1^·Pa^–1^), owing to an enhanced non-thermal effect of single-mode microwave heating and balanced dissolution rate of TiS_2_. Additionally, Ti_8_-oxo ([Ti_8_(μ_2_-O)_8_(OOCC_6_H_5_)_16_]) [162] and Ti_6_-oxo ([Ti_6_O_6_(abz)_6_(OiPr)_6_](iPrOH)_n_, abz = 4-aminobenzoic acid) clusters [163] were further investigated as a titanium source combining single-mode microwave heating for the tertiary growth of MIL-125 membranes. Employing the Ti_8_ cluster source led to not only a lower reaction temperature required for the formation of a well-intergrown MIL-125 membrane but also linker deficiencies within the framework, resulting in enhanced CO_2_/N_2_ selectivity. The fast and stable release of Ti(IV) ions from the Ti_6_-oxo cluster resulted in the more precise control of the growth kinetics of seed layers and, therefore, the formation of well-intergrown preferentially c-oriented MIL-125(Ti) membranes.

To avoid undesired crystal twinning during the epitaxial growth of the NH_2_-MIL-125 seed layer, Sun et al. [160] developed a facile competitive metal ion-based coordination modulation strategy by the simple addition of a wide range of metal salts (e.g., Cu(NO_3_)_2_, Co(NO_3_)_2_, and Zn(NO_3_)_2_) in the precursor solution. The competitive coordination of NH_2_-BDC ligands between competitive metal ions and Ti_8_-oxo clusters enabled the moderate equilibrium concentration of NH_2_-MIL-125 secondary building units, thereby inhibiting the bulk NH_2_-MIL-125 nucleation with no compromise in the lateral intergrowth of the seed layer (Figure 9c).

Recently, Sun et al. [161] explored the preparation of highly *c*-oriented sub-100 nm-thick NH_2_-MIL-125 membranes chelated with coordinatively unsaturated Cu ions (denoted as Cu@ORI-MIL-M) by the epitaxial growth of ultrathin Cu@NH_2_MIL-125 nanosheet seeds (Figure 9d). Employing copper acetylacetonate (Cu(acac)_2_) as the growth modulator was found to be crucial for the formation of ultrathin Cu@NH_2_MIL-125 nanosheets and the coordinatively unsaturated Cu ions anchored by the –NH_2_ facilitated π-complexation interactions with C_2_H_4_, resulting in an C_2_H_4_/C_2_H_6_ selectivity approaching 13.6 and CO_2_/N_2_ selectivity of 43.2 with a CO_2_ permeance of 696 GPU.

## 9. Conclusions and Outlook

A periodic network and distinct pore size within pure MOF membranes is expected to promote the fast diffusion through the membrane, leading to simultaneously enhanced permeability and selectivity. The unique properties make them promising candidates for applications in gas separation, seawater desalination, organic solvent dehydration, and more. Different types of pure MOF membranes have been fabricated on the substrates including imidazolate-based ZIFs, multi-dentated carboxylate-based Zr-MOFs, MILs, and HKUST-1. This review has summarized various preparation methods for MOF membranes of different metal species, including in situ solvothermal synthesis, secondary growth, electrochemical deposition, counter diffusion growth, liquid phase epitaxy, and solvent-free synthesis, and pointed out the advantages and disadvantages of each method. Moreover, the history and state-of-the-art advances of different preparation methods for each type of MOF membrane were summarized in detail, providing a reference for the selection of preparation methods.

The research on MOF membranes has made significant progress, leading to notable improvements in their preparation methods and expanded applications across different fields. Despite these advancements, challenges persist in the preparation of pure MOF membranes for future industrial applications in terms of mechanical stability, processibility, scalability, and reproducibility. Although extensive research has been conducted, most pure MOF membrane fabrication methods are still far away from meeting the industrialization requirements, primarily due to heavy costs and arguable stability. This review outlines the following potential directions for the future development of the fabrication of polycrystalline pure MOF membranes. Unlike bulk MOF crystals, the crystallization kinetics of pure MOF membranes have been rarely investigated. However, understanding the formation process of pure MOF membranes from nucleation to crystallinity is of great significance for their mechanical stability, continuity, orientated growth, and separative performance for all the explored pure MOF membrane fabrication methods. In addition, the assembly of MOF networks can be susceptible to various synthetic environmental factors, such as temperature, pressure, solvent, concentration, additives, etc. Elucidating the influence of these environmental factors on the pure membrane growth kinetics is crucial for the quality-controlled reproducible fabrication of membranes. Regarding in situ solvothermal synthesis, increasing the nucleation sites on the surface of the substrates is key to preparing continuous and well-intergrown membranes with improved mechanical strength. For secondary growth, the seed layer is crucial for the membrane growth. As far as preferred orientation control is concerned, uniform MOF nanosheets are considered as ideal seed candidates because of the ease of deposition, better thickness control, and versatile functionality [92]. Although electrochemical deposition can achieve the controllable thickness and crystal size of MOF membranes, research on controlling the orientation and defects is not yet in-depth [46]. Counter diffusion synthesis has been applied on limited MOFs under mild reaction conditions. Therefore, it is necessary to study the applicability of more types of MOF membranes for counter diffusion synthesis [50]. ZIFs, Cu-MOFs, and Zr-based MOF films have been prepared using the LPE method, which could consume substantial solvents. Future research should focus on expanding the application scope of the LPE method with less reagent consumption for industrial consideration. Reduce the cost of MOF membrane preparation or reducing solvent usage through solvent-free synthesis are crucial strategies. Solvent-free synthesis is a promising method for preparing MOF membranes, as it can avoid the high costs of reagents and environmental pollution caused by large amounts of solvents. However, it has been limited to few MOFs (e.g., ZIF-8 and HKUST-1). Developing improved solvent-free strategies are demanding for the solvent-free fabrication of other pure MOF membranes [164]. In addition to these traditional preparation methods, new technologies such as the microfluidic approach have emerged, making the preparation of MOF membranes more efficient [165]. Although there are still various challenges in the application of MOF membranes, their vast structural diversity allows for the utilization of an increasing number of MOFs to create high-performance MOF membranes. Recent developments in atomic simulations and artificial intelligence methods have further enhanced this potential, indicating a promising outlook for their broad applications in the future [166].
